# Distress in parents of children with first-onset steroid-sensitive nephrotic syndrome

**DOI:** 10.1007/s00467-023-06038-1

**Published:** 2023-06-28

**Authors:** Floor Veltkamp, Hedy A. van Oers, Lorynn Teela, Elske M. Mak-Nienhuis, Lotte Haverman, Antonia H. M. Bouts, on behalf of the LEARNS consortium, Anna Bael, Antonia H. M. Bouts, Nynke Buter, Hans van der Deure, Eiske Dorresteijn, Sandrine Florquin, Valentina Gracchi, Flore Engels, Francis Kloosterman-Eijgenraam, Elena Levtchenko, Elske Mak-Nienhuis, Ron Mathôt, Floor Oversteege, Saskia de Pont, Roos van Rooij-Kouwenhoven, Michiel Schreuder, Rixt Schriemer, Paul Vos, Johan Vande Walle, Joanna van Wijk

**Affiliations:** 1 Department of Pediatric Nephrology, Emma Children’s Hospital, Amsterdam University Medical Centers, Location University of Amsterdam, Meibergdreef 9, Amsterdam, The Netherlands; 2 Child Development, Amsterdam Reproduction and Development, Amsterdam, The Netherlands; 3 Child and Adolescent Psychiatry & Psychosocial Care, Emma Children’s Hospital, Amsterdam University Medical Centers, Location University of Amsterdam, Meibergdreef 9, Amsterdam, The Netherlands; 4 Mental Health, Amsterdam Public Health, Amsterdam, The Netherlands; 5 Department of Pediatric Nephrology, Emma Children’s Hospital, Amsterdam University Medical Centers, University of Amsterdam, Post Box, 22660, 1100 DD Amsterdam, The Netherlands

**Keywords:** Steroid-sensitive nephrotic syndrome, First onset, Parental distress

## Abstract

**Background:**

Steroid-sensitive nephrotic syndrome (SSNS) is associated with a relapsing–remitting course that can be stressful for parents. As little is known of parental distress at the first onset of SSNS, this study aims to describe parental distress and everyday problems in mothers and fathers of a child with newly diagnosed SSNS participating in a randomized controlled trial of levamisole added to corticosteroids.

**Methods:**

To assess distress, the Distress Thermometer for Parents (DT-P) was used, which includes questions on distress (thermometer score 0–10, ≥ 4 “clinical distress”) and presence of everyday problems in six domains: practical, social, emotional, physical, cognitive, and parenting. The DT-P was completed 4 weeks after the onset of SSNS. Total sum and individual items of everyday problems were compared with reference data from mothers and fathers of the Dutch general population.

**Results:**

There was no difference in clinically elevated parental distress between SSNS mothers (*n* = 37) and fathers (*n* = 25) and reference parents. Compared to reference fathers, fathers of a child with SSNS scored significantly higher on emotional problems (*P* = 0.030), while mothers experienced more parenting problems (*P* = 0.002). Regression analyses showed that lower parental age and having a girl with SSNS were significantly associated with more practical problems and higher distress thermometer scores, respectively.

**Conclusions:**

Four weeks after onset, SSNS mothers and fathers experience equal distress as reference parents. However, both parents endorsed significantly more everyday problems. Therefore, monitoring parental distress, even in the first weeks of the disease, could contribute to timely interventions and prevent worsening of problems.

**Clinical trial registry:**

Dutch Trial Register (https://onderzoekmetmensen.nl/en/trial/27331).

**Graphical abstract:**

**Figure:**
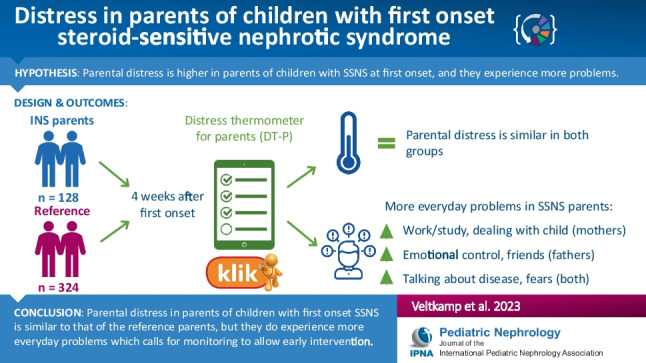
A higher resolution version of the Graphical abstract is available as Supplementary information

**Supplementary information:**

The online version contains supplementary material available at 10.1007/s00467-023-06038-1.

## Introduction

Idiopathic nephrotic syndrome is a rare disease of childhood characterized by profound edema, severe proteinuria, hypoalbuminemia, and hyperlipidemia [1, 2]. Incidence peaks between the age of 2 and 6 years and is higher in boys than in girls. Following initial treatment with high-dose corticosteroids, the vast majority (80–90%) achieve remission (steroid-sensitive nephrotic syndrome (SSNS)). However, around 70–80% of the patients experience at least one relapse of the disease and one-half of them develop frequently relapsing nephrotic syndrome (FRNS) or steroid-dependent nephrotic syndrome (SDNS) [3]. Relapses require repeated courses of corticosteroids, but since steroid use is associated with marked side effects, steroid-sparing drugs are often introduced. Still, these drugs are also not without side effects.

At the first onset, parents are confronted with their previously healthy child being ill and — often, but not always — admitted to a hospital. Such medical events are moderately associated with post-traumatic stress disorder in adults [4]. Today, clinicians are still unable to predict which child will relapse and, if so, how often. This relapsing–remitting course makes SSNS a chronic illness. In parents of children with a chronic illness, it is known that the child’s disease leads to significantly greater parental stress, which poses a greater risk for psychosocial problems, like lower health-related quality of life (HRQoL) [5], post-traumatic stress disorder [6, 7], and distress [7–9]. In turn, parental distress influences the well-being of the child [10]. Concerning SSNS, only a limited number of studies have been conducted to study parental distress in parents with a child with SSNS [11–14]. It is reported that parents experience a significant burden and psychological distress, and show more symptoms of depression than the reference groups. However, these studies were conducted among parents of children with FRNS or SDNS and at different disease stages (remission versus active disease). To date, the level of distress in parents of a child with first-onset SSNS is unknown.

In order to understand the impact of their child’s diagnosis of SSNS on parents, we conducted an exploratory, cross-sectional study to determine whether parental distress and everyday problems are more frequent in fathers and mothers whose child was recently diagnosed with SSNS than in those without SSNS. Additionally, we assessed which sociodemographic and/or clinical variables were associated with parental distress and everyday problems.

## Methods

### Study design

This is a prospective, cross-sectional study that is part of the LEARNS randomized, placebo-controlled trial that studies the efficacy of additional levamisole to corticosteroids to prevent relapses in children with first-onset SSNS [15]. Children were followed for a total duration of 2 years.

Parent-reported outcomes measures (ParROMs) — questionnaires that assess parental functioning — were completed by using the KLIK PROM portal (www.hetklikt.nu) [16]. At registration, parents were asked for an additional informed consent after which the ParROMs were available for completion. Automatic reminders were sent after 7 days. During follow-up, ParROMs were collected at randomization before the start of study medication (week 4), after discontinuation of study medication (week 28), at primary endpoint (year 1), and at the end of study (year 2). For this study, only the ParROM collected at randomization (week 4) was used to evaluate parental distress at first-onset SSNS.

### Patient and parent selection

Children between 2 and 16 years with first-onset SSNS were eligible for participation in the randomized controlled trial (RCT). The remaining inclusion and exclusion criteria have been described in detail elsewhere [15]. Both mothers and fathers were invited to complete the Distress Thermometer for Parents (DT-P).

### Measures

#### Parental and child characteristics

One parent of the child completed the general sociodemographic questionnaire about age, sex, marital status (married/living together or divorced/separated/widowed), number of children living at home (1, 2, or 3 or more), educational level (low [primary education, lower vocational education, lower or middle general secondary education], intermediate [middle vocational education, higher secondary education, pre-university education], or high [higher vocational education, university]), and occupational status (paid employment or not) (of both self and that of partner). Clinical data of the children (age and sex) were prospectively collected by trained clinicians using a standardized electronic case report form (Castor EDC).

#### Distress thermometer for parents

To measure perceived parental distress and everyday problems, parents completed the DT-P [9]. The version for parents of children of ≥ 2 years consists of three parts: (1) a thermometer to indicate the overall distress in the past week ranging from 0 (no distress) to 10 (extreme distress), scores of ≥ 4 indicate clinically elevated distress; (2) a 34-item problem list covering six domains (practical (*n* = 7), family/social (*n* = 4), emotional (*n* = 9), physical (*n* = 7), cognitive (*n* = 2), and parenting (*n* = 5)); and (3) five additional questions about perceived support and wish for referral [9]. The items in the problem list were scored as either problematic (Yes = 1) or not (No = 0). Two total problem scores were calculated: the sum of all problem domains (1) without parenting (range 0–29) and (2) including parenting (range 0–34). Cronbach’s alpha of the domains ranged from 0.169 to 0.836 and from 0.592 to 1.000 in fathers and mothers of our sample, respectively (Online Resource 1 Table S1). Sum scores of domains with *α* < 0.50 were not presented due to low internal consistency (practical problems, social problems, and cognitive problems in fathers). Reference data of parents from the Dutch general population were available [17]. To obtain a representative reference group, only parents with a child aged between 2 and 16 years were selected.

### Statistical analyses

Continuous data were presented as means (standard deviation (SD)) or median (range), depending on distribution. Normality of the data was checked through histograms and QQ plots. For continuous data, we used an independent *t*-test or the Mann–Whitney *U* test for normally or non-normally distributed data, respectively. Discrete data were presented as frequencies and proportions and compared using Fisher’s exact test. To quantify differences, effect sizes (Cohen’s *d* for parametric data, *r* for non-parametric data) or odds ratios (ORs) with 95% confidence intervals (CIs) were calculated. Cohen’s *d* was calculated as the difference in means divided by the pooled standard deviation, while *r* was calculated as the *z*-score divided by the square root of the total population ($r =\frac{z}{\sqrt{N}})$. An effect size of 0.2, 0.5, or 0.8 was considered small, moderate, or large, respectively [18].

Thermometer scores, domain scores, item scores, and additional questions of mothers and fathers of children with SSNS were compared to scores of reference mothers and fathers, respectively. In the case of baseline differences, the association with the thermometer score was tested by Pearson’s coefficient. If significant, an ANOVA (continuous data) or the Mantel–Haenszel test (discrete data) was used to correct for confounders.

To identify which sociodemographic and clinical variables (Online Resource 1 Table S2) were associated with thermometer and domain scores, we conducted linear regression analyses. First, a univariate regression analysis was performed. Variables (parental age, mother/father, born in the Netherlands or Belgium, education, paid employment, number of children, and the child’s age and sex) that obtained a *P*-value < 0.05 in at least one of the problem domains were included simultaneously in the multiple linear regression model (child’s sex and parental age). Following the univariate analysis (Online Resource 1 Table S3), the child’s sex and parental age were included in the multiple regression analysis. Correlation between the variables was tested for multicollinearity. A correlation of > 0.8 was considered too high. None of the variables was excluded from the final model. To express the association between variables and outcome, standardized regression coefficients (*β*) were calculated. For continuous variables, *β* were considered small if 0.1, medium if 0.3, and large if 0.5, while for dichotomous variables, *β* were considered small if 0.2, medium if 0.5, and large if 0.8 [18].

For all statistical analyses, IBM SPSS Statistics [19] and R Studio [20] were used. A *P-*value of < 0.05 was considered statistically different.

### Ethical considerations

The LEARNS study was conducted in accordance with the Declaration of Helsinki. Institutional review boards in and the competent authorities of both countries approved the study protocol. The LEARNS study is registered with the Dutch Trial Register (https://onderzoekmetmensen.nl/en/trial/27331). Written informed consent and/or assent (if appropriate) was obtained from the parent(s) and the child (≥ 12 years).

## Results

In total, parents of 46 children with SSNS were eligible and 62 parents (37 mothers, 25 fathers) of 37/46 (80%) children completed at least one questionnaire. Eight families consisted of a single parent. A total of 46 patients (37 mothers and 25 fathers) were included in the RCT and completed the DT-P at baseline (week 4), corresponding to a response rate of 80%. Table 1 displays the baseline characteristics of the children and their parents. Both mothers (70.3% vs. 96.5%, *P* < 0.001) and fathers (92.0% vs. 94.9%, *P* = 0.004) were less often born in the Netherlands or Belgium than reference parents. Fathers were more often living separately from the mother than reference fathers (12.0% vs. 2.6%, *P* = 0.037).

**Table 1:** Sociodemographic characteristics of parents of children with SSNS and parents of a non-chronically ill child (reference group)

	Mothers			Fathers		
	SSNS (*N* = 37)	Reference (*N* = 623)	*P*	SSNS (*N* = 25)	Reference (*N* = 391)	*P*
Parents						
Age, mean (SD)	37.5 (6.14)	39.3 (5.67)	0.09	41.4 (6.64)	42.3 (6.70)	0.52
Born in the Netherlands or Belgium, *n* (%)^a^	26 (70.3)	601 (96.5)	** < 0.001**	23 (92.0)	371 (94.9)	0.63
Educational level, *n* (%)			0.08			0.48
Low	4 (10.8)	94 (15.1)		2 (8.0)	68 (17.4)	
Intermediate	12 (32.4)	297 (47.7)		11 (44.0)	168 (43.0)	
High	20 (54.1)	230 (36.9)		11 (44.0)	149 (38.1)	
Paid employment, *n* (%)	30 (81.1)	486 (78.0)	0.84	25 (100)	362 (92.6)	0.40
Marital status, *n* (%)^a^			0.21			**0.037**
Married/living together	29 (78.4)	540 (86.7)		22 (88.0)	381 (97.4)	
Separated/single parent/widowed	8 (21.6)	83 (13.3)		3 (12.0)	10 (2.6)	
Children living at home			0.55			0.46
1	5 (13.5)	103 (16.5)		4 (16.0)	53 (13.6)	
2	20 (54.1)	367 (58.9)		12 (48.0)	236 (60.4)	
3 or more	12 (32.4)	153 (24.6)		9 (36.0)	102 (26.1)	
Child						
Age^b^, median (range)	6.4 (2.6–15.5)	7.4 (2.0–15.9)	0.18^c^	6.8 (3.0–14.8)	7.4 (2.0–15.9)	0.44^c^
Male, *n* (%)	23 (62.2)	326 (52.3)	0.31	16 (64.0)	217 (55.5)	0.53

*SD*, standard deviation; *SSNS*, steroid-sensitive nephrotic syndrome

^a^The Pearson coefficient indicated that country of birth (mothers) had no influence on distress, but marital status (fathers) did (*r* = 0.11, *P* = 0.037). Therefore, correction took place for the latter

^b^Age at the first onset of SSNS

^c^By Mann–Whitney *U* test

Significant *P*-values (<0.05) are shown in bold

### Parental distress

The mean ± SD thermometer score of mothers and fathers with a child with SSNS was 3.8 ± 2.8 and 3.6 ± 2.7, respectively, which corresponded with a clinical score (≥ 4) of 48.6% and 52.0%. This was not significantly different from reference parents (Table 2). Where country of birth was not associated with the thermometer score, marital status (fathers) was (*r* = 0.11, *P* = 0.037) and was corrected for, accordingly.

**Table 2:** Parenting distress in mothers and fathers of a child with SSNS compared to reference parents

	Mothers					Fathers				
	SSNS (*N* = 37)	Reference (*N* = 623)	*P*	OR/*d*	95% CI	SSNS (*N* = 25)	Reference (*N* = 391)	*P*	OR/*d*	95% CI
Thermometer score, mean SD	3.81 (2.75)	3.71 (2.75)	0.83	0.04	− 0.29 to 0.37	3.64 (2.64)	3.07 (2.65)	0.17	0.22	− 0.18 to 0.62
Clinical score, *n* (%)	18 (48.6)	280 (44.9)	0.73	1.16	0.56 to 2.39	13 (52.0)	140 (35.8)	0.24	0.55	0.24 to 1.27
Practical problems, mean (SD)	1.35 (1.58)	1.12 (1.36)	0.39	0.17	− 0.16 to 0.50	–^a^	–	–	–	–
Housing, *n* (%)	2 (5.4)	40 (6.4)	> 0.99	0.83	0.09 to 3.45	1 (4.0)	14 (3.6)	0.73	1.56	0.14 to 17.1
Work/study, *n* (%)	15 (40.5)	143 (23.0)	**0.027**	2.28	1.07 to 4.75	5 (20.0)	94 (24.0)	> 0.99	1.25	0.26 to 6.74
Finances/insurance, *n* (%)	1 (2.7)	107 (17.2)	**0.020**	0.13	0.00 to 0.82	3 (12.0)	57 (14.6)	0.86	1.33	0.39 to 4.52
Housekeeping, *n* (%)	11 (29.7)	146 (23.4)	0.43	1.38	0.60 to 2.98	2 (8.0)	47 (12.0)	0.66	1.72	0.40 to 7.35
Transport, *n* (%)	2 (5.4)	40 (6.4)	> 0.99	0.83	0.09 to 3.45	3 (12.0)	19 (4.9)	0.31	0.40	0.11 to 1.45
Child care/supervision, *n* (%)	7 (18.9)	72 (11.6)	0.19	1.78	0.64 to 4.34	3 (12.0)	25 (6.4)	0.51	0.51	0.14 to 1.83
Leisure activities/relaxing, *n* (%)	12 (32.4)	150 (24.1)	0.24	1.51	0.68 to 3.21	6 (24.0)	63 (16.1)	0.64	0.51	0.25 to 1.66
Social problems, mean (SD)	0.38 (0.89)	0.44 (0.80)	0.68	− 0.07	− 0.40 to 0.26	–^a^	–	–	–	–
Dealing with (ex)partner, *n* (%)	4 (10.8)	87 (14.0)	0.81	0.75	0.19 to 2.18	2 (8.0)	42 (10.7)	0.87	1.45	0.33 to 6.27
Dealing with family, *n* (%)	3 (8.1)	70 (11.2)	0.79	0.70	0.13 to 2.31	1 (4.0)	26 (6.6)	0.68	1.54	0.20 to 11.9
Dealing with friends, *n* (%)	1 (2.7)	27 (4.3)	> 0.99	0.61	0.01 to 3.95	5 (20.0)	7 (1.8)	** < 0.001**	**0.12**	**0.03 to 0.47**
Interacting with your child(ren), *n* (%)	6 (16.2)	90 (14.4)	0.81	1.15	0.38 to 2.90	2 (8.0)	44 (11.3)	0.65	1.72	0.41 to 7.24
Emotional problems, mean (SD)	2.05 (2.04)	1.83 (2.14)	0.52	− 0.10	− 0.43 to 0.23	2.12 (2.07)	1.14 (1.71)	0.57	0.57	− 0.16 to 0.98
Keeping emotions under control, *n* (%)	12 (32.4)	178 (28.6)	0.58	1.20	0.54 to 2.54	11 (44.0)	57 (14.6)	**0.002**	**0.27**	**0.12 to 0.64**
Self-confidence, *n* (%)	2 (5.4)	147 (23.6)	**0.008**	0.18	0.02 to 0.74	2 (8.0)	51 (13.0)	0.65	1.76	0.41 to 7.59
Fears, *n* (%)	11 (29.7)	65 (10.4)	**0.002**	3.62	1.54 to 8.02	6 (24.0)	23 (5.9)	**0.002**	**0.19**	**0.07 to 0.52**
Depression, *n* (%)	11 (29.7)	208 (33.4)	0.72	0.84	0.37 to 1.81	10 (40.0)	92 (23.5)	0.12	0.47	0.20 to 1.09
Feeling tense or nervous, *n* (%)	17 (45.9)	227 (36.4)	0.29	1.48	0.71 to 3.05	12 (48.0)	112 (28.6)	0.08	0.44	0.20 to 1.01
Loneliness, *n* (%)	6 (16.2)	55 (8.8)	0.14	2.00	0.65 to 5.15	3 (12.0)	19 (4.9)	0.62	0.60	0.17 to 2.13
Feeling of guilt, *n* (%)	5 (13.5)	110 (17.7)	0.66	0.73	0.22 to 1.94	4 (16.0)	29 (7.4)	0.17	0.37	0.12 to 1.17
Use of substances, *n* (%)	0 (0)	17 (2.7)	0.61	0.00	0.00 to 4.16	0 (0)	8 (2.0)	0.88	–	–
Intrusive/recurrent thoughts	12 (32.4)	134 (21.5)	0.15	1.75	0.78 to 3.73	5 (20.0)	53 (13.6)	0.88	0.90	0.26 to 3.11
Physical problems, mean (SD)	1.35 (1.38)	1.79 (1.72)	0.07	− 0.26	− 0.07 to 0.59	1.36 (1.44)	1.28 (1.45)	0.32	0.06	− 0.34 to 0.46
Eating, *n* (%)	3 (8.1)	76 (12.2)	0.61	0.64	0.12 to 2.10	1 (4.0)	17 (4.3)	0.88	1.18	0.15 to 9.05
Weight, *n* (%)	4 (10.8)	171 (27.4)	**0.033**	**0.32**	**0.08 to 0.92**	4 (16.0)	56 (14.3)	0.85	1.12	0.35 to 3.52
Sleep, *n* (%)	15 (40.5)	169 (27.1)	0.09	1.83	0.86 to 3.79	10 (40.0)	83 (21.2)	0.08	0.43	0.18 to 1.00
Fatigue, *n* (%)	18 (48.6)	337 (54.1)	0.61	0.80	0.39 to 1.65	11 (44.0)	170 (43.5)	0.95	1.12	0.49 to 2.60
Out of shape/condition, *n* (%)	6 (16.2)	145 (23.3)	0.42	0.64	0.21 to 1.59	4 (16.0)	72 (18.4)	0.78	1.33	0.45 to 3.89
Pain, *n* (%)	3 (8.1)	154 (24.7)	**0.017**	**0.27**	**0.05 to 0.87**	3 (12.0)	67 (17.1)	0.64	1.57	0.46 to 5.33
Sexuality, *n* (%)	1 (2.7)	66 (10.6)	0.16	0.23	0.01 to 1.44	1 (4.0)	36 (9.2)	0.61	2.39	0.32 to 18.1
Cognitive problems, mean (SD)	0.62 (0.86)	0.43 (0.73)	0.19	0.26	− 0.07 to 0.59	–^a^	–	–	–	–
Concentration, *n* (%)	11 (29.7)	120 (19.3)	0.13	1.77	0.77 to 3.84	5 (20.0)	47 (12.0)	0.56	0.62	0.22 to 1.79
Memory, *n* (%)	12 (32.4)	148 (23.8)	0.24	1.54	0.69 to 3.27	6 (24.0)	56 (14.3)	0.48	0.60	0.22 to 1.63
Parenting problems ≥ 2 years, mean (SD)^b^	0.77 (1.06)	0.42 (0.88)	**0.002**^**c**^	0.09^d^	− 0.45 to 0.63	0.40 (0.82)	0.35 (0.87)	0.24	0.01^d^	− 0.34 to 0.36
Dealing with your child, *n* (%)	4 (10.8)	81 (13.0)	> 0.99	0.85	0.21 to 2.50	2 (8.0)	40 (10.2)	0.89	1.41	0.33 to 6.13
Dealing with the feelings of your child, *n* (%)	10 (27.0)	72 (11.6)	**0.008**	**3.02**	**1.24 to 6.83**	4 (16.0)	38 (9.7)	0.68	0.67	0.22 to 2.03
Talking about the disease/consequences with your child, *n* (%)	6 (16.2)	24 (3.9)	**0.004**	**5.08**	**1.58 to 14.1**	3 (12.0)	10 (2.6)	**0.030**	**0.18**	**0.04 to 0.69**
Independence of your child, *n* (%)	5 (13.5)	55 (8.8)	0.36	1.70	0.49 to 4.67	1 (4.0)	30 (7.7)	0.75	2.05	0.27 to 5.05
Following advice about treatment/giving medication, *n* (%)	2 (5.4)	27 (4.3)	0.66	1.32	0.15 to 5.66	0 (0)	15 (3.8)	0.70	–	–
Total score (5 domains), mean (SD)	5.76 (5.28)	5.62 (5.25)	0.88	0.03	− 0.30 to 0.36	5.24 (4.49)	3.80 (4.28)	0.33	0.34	to 0.07 to 0.75
Total score (6 domains), mean (SD)	6.74 (5.85)	6.04 (5.71)	0.49^c^	0.01^d^	− 0.51 to 0.53	5.64 (4.87)	4.13 (4.74)	0.27	0.05^d^	− 0.37 to 0.47
Additional questions										
Enough support from surroundings, *n* (%)	35 (94.6)	560 (89.9)	0.57	1.97	0.49 to 17.3	24 (96.0)	360 (92.1)	0.65	0.44	0.06 to 3.29
People react with lack of understanding, *n* (%)	5 (13.5)	87 (14.0)	> 0.99	0.96	0.29 to 2.58	1 (4.0)	48 (12.3)	0.33	3.44	0.46 to 25.5
Would like to talk to a professional about situation, *n* (%)	12 (32.4)	114 (18.3)	**0.0496**	2.14	0.95 to 4.58	5 (20.0)	60 (15.3)	0.89	0.81	0.30 to 2.22

^a^Scores not presented due to low internal consistency (Cronbach’s *α* < 0.500)

^b^Parents of a child ≥ 2 years of age wrongfully indicated that their child was < 2 years of age. As a result, 2, 7, and 10 parents from the SSNS mothers, reference mothers, and reference fathers did not complete the domain Parenting problems for children ≥ 2 years of age

^c^By Mann–Whitney *U* test

^d^Effect size (*r*) for non-normally distributed data

Significant *P*-values (<0.05) are shown in bold

Compared to reference mothers, mothers with a child with SSNS scored significantly higher on parenting problems (0.8 ± 1.1 vs. 0.4 ± 0.9, *P* = 0.002, *d* = 0.09), but not on any other domain.

Regarding everyday problems, mothers reported significantly more problems on 4/34 items (Table 2) compared to reference mothers. Mothers of a child with SSNS had significantly more problems with work or study (40.5% vs. 23.0%, *P* = 0.027), fears (29.7% vs. 10.4%, *P* = 0.008), dealing with their child (27.0% vs. 11.6%, *P* = 0.008), and talking about the disease (16.2% vs. 3.9%, *P* = 0.004). At the same time, also fewer problems were reported in 4/34 items (finances, pain, weight, and self-confidence) (Table 2) compared to reference mothers.

There were no differences in the domain scores between fathers of an SSNS child and reference fathers. Compared to reference fathers, fathers of an SSNS child reported significantly more problems on 4/34 items: dealing with friends (20.0% vs. 1.8%, *P* < 0.001), keeping emotions under control (44.0% vs. 14.6%, *P* = 0.002), fears (24.0% vs. 5.9%, *P* = 0.002), and talking about the disease (12.0% vs. 2.6%, *P* = 0.039).

### Additional questions

When asked about support, there was no difference in people reacting with a lack of understanding or having enough support from their surroundings between parents of a child with SSNS and reference parents. However, mothers of an SSNS child indicated more often that they wished to talk with a professional about the situation than the reference group (32.4% vs. 18.3%, *P* = 0.0496) (Table 2).

### Variables associated with parental distress

Multiple regression analysis showed that the child’s sex and parental age had a significant association with the thermometer score and practical problems, respectively. Parents of a girl with SSNS (*β* = 0.26, *P* = 0.045) had significantly higher thermometer scores than parents of a boy with SNSS, while lower parental age was significantly associated with more practical problems (*β* = 0.29, *P* = 0.020) (Table 3).

**Table 3:** Results from the multiple regression analysis. Variables that obtained significance levels *P* < 0.05 in the univariate analysis were included in the final model. Standardized regression coefficients (*β*) were calculated to express the explained variance of each variable

	Thermometer score		Practical problems		Social problems		Emotional problems		Physical problems		Cognitive problems		Parenting problems		Total (5 domains)		Total (6 domains)	
	*β*	*P*	*β*	*P*	*β*	*P*	*β*	*P*	*β*	*P*	*β*	*P*	*β*	*P*	*β*	*P*	*β*	*P*
Age parent	− 0.11	0.37	− 0.29	**0.020**	− 0.18	0.17	0.05	0.73	− 0.08	0.56	− 0.21	0.10	− 0.21	0.10	− 0.15	0.24	0.07	0.66
Male child	− 0.26	**0.045**	− 0.07	0.55	− 0.04	0.21	− 0.15	0.54	0.08	0.53	− 0.05	0.20	0.06	0.67	− 0.08	0.56	− 0.16	0.33
Adjusted *R*^2^	0.05		0.07		0.00		− 0.01		− 0.02		0.02		0.01		0.00		− 0.02	
*F*-statistic	2.63	0.08	3.159	0.0497	1.046	0.36	0.75	0.48	0.35	0.71	1.50	0.23	1.43	0.25	0.91	0.41	0.58	0.57

Significant *P*-values (<0.05) are shown in bold

## Discussion

This is the first study that systematically investigated parental distress at the first presentation of SSNS, showing the results of a psychosocial screening questionnaire for distress and the presence of everyday problems in parents of a child with SSNS compared to a group of reference parents. Clinically elevated distress was not more frequently observed in parents of children with SSNS compared to the reference group. However, mothers reported more parenting problems than reference mothers. Additionally, having more problems with work or study, dealing with their child (mothers), keeping emotions under control, dealing with friends (fathers), and fears, and talking about the disease (both) were reported. Being a parent of a girl with SSNS was associated with higher stress thermometer scores, and lower parental age with more practical problems, which was not the case in reference parents.

In contrast to other studies that focused on parents with a child with FRNS/SDNS [11, 12], there was not a greater risk for clinically elevated parental distress in this study. This may be partly explained by the fact that the children in our study were in remission. For example, in the study by Esezobor et al., higher parental distress was observed, but their children were in relapse [13]. Also, we studied parental distress after the first onset instead of relapsing SSNS. However, direct comparison of the studies is complicated by (1) the use of different ParROMs (Beck’s Depression Inventory, PedsQL Family Impact Module, Zarit Burden Interview), (2) inadequate reference population, (3) conducted many years ago [14], or (4) conducted in non-Western countries (India [11, 12] and Nigeria [13]). In the latter, housing, financial, healthcare, and family situations are different from Western countries. Last, our parents are part of a well-organized trial and, therefore, may have experienced more attention or focused care that could have lowered their distress.

SSNS is characterized by its relapsing–remitting pattern. Despite many clinical trials investigating different courses of corticosteroids, the risk for relapse after the first onset has been around 70–80% for decades [21]. To date, no biomarkers that could predict prognosis after the first onset have been identified. Clinicians are still unable to tell which child is at greater risk for relapse. This uncertainty may be reflected in the finding that both parents reported more problems with fears than reference parents. Additionally, at 4 weeks after the first onset, side effects of corticosteroids peak in children with SSNS. Steroid-related mood changes and behavioral problems were frequently reported in our study population, which affected the psychosocial functioning of these children in terms of emotional and behavioral difficulties (Veltkamp et al., manuscript submitted but unpublished to date). This may impact the family’s day-to-day lives, leading to parental distress, as is shown by more parenting problems in mothers.

Our results suggest that the care for the child relies more heavily on mothers than on fathers — or that mothers feel that they should take care more than fathers. This is substantiated by the finding that mothers of a child with SSNS had more problems with work or study than reference mothers, while fathers did not. A previous study has shown that maternal labor force participation (working > 12 h/week) is lower in families with a child with a chronic illness [22]. However, recent studies using the DT-P did not find a difference in work-related problems [23–28]. These studies included mothers and fathers of children with inflammatory bowel disease [27], avoidant restrictive food disorder [24], home parental nutrition [26], Down syndrome [25], Marfan syndrome [28], and mucopolysaccharidosis type 3 [23]. Within 4 weeks, some mothers of a child with SSNS were unable to adapt to this relatively new situation, while mothers of a child with other chronic illnesses — that may have been present even from birth — may have had more time to adjust and settle things with work (e.g., working fewer hours). Although in our study the hours of paid work are unknown (anything between 12 and 40 h per week), comparable proportions of mothers in our and previous studies had paid employment [23–25, 27, 28]. This further stresses that time is more likely to be a factor in first-onset SSNS.

In addition to work problems, parenting problems were more prevalent in SSNS mothers, which was mainly the result of having more problems with dealing with the feelings of and talking about the disease with their child. Parenting problems were also observed in previous studies with parents of children with inflammatory bowel disease, mucopolysaccharidosis type II, and home parental nutrition [23, 26, 27]. Although fathers also reported more problems with talking about the disease, they did not experience more problems with overall parenting. However, fathers reported more problems with keeping emotions under control, fears, dealing with friends, and talking about the disease than reference fathers in the current study. This shows that fathers do struggle with their child being ill. On the other hand, roughly one-third of the mothers with a child with SSNS indicated that they would like to talk to a professional about their situation, which was not the case for fathers.

In previous studies, country of birth and paid employment have been associated with higher scores on parental distress and more everyday problems. Apart from a female child and lower parental age, no sociodemographic or child characteristics (age) were associated with parental distress. Higher thermometer scores (i.e., more parental distress) were reported in parents of girls with SSNS. Whether this is because parents worry more about their daughters, or that girls display more problematic behavior — steroid-related or not, is not known. Younger parents may be less experienced in parenting without a stable financial and/or family situation, leading to more practical problems. Also, younger parents presumably have younger child(ren) who are more dependent on their care. Clinicians should be aware that younger parents may be more prone to distress. However, it must be noted that the association for both variables was small to moderate, even for psychosocial studies.

There are some limitations to this study. First, there may have been a selection bias as the inclusion rate of participation in the RCT is around 55–60% (inclusions not yet finished). Main reasons for exclusion and declination were not willing to participate (traveling distance to participating hospital, time consuming, faith in good outcome), language barrier, or steroid-resistant nephrotic syndrome. Parents who participated in the trial may have been more motivated and higher educated, and have a more stable family situation. This could have skewed the results. Moreover, parents may have been more hopeful for a better outcome as their child had a 50% chance to have received levamisole, which is thought to be more efficacious in preventing relapses of SSNS. Second, although the response rate was high, our sample size was relatively small. This could have limited the statistical power to detect meaningful differences between our sample and the reference group. Third, given that we compared 34 different outcomes per group, there is a chance of 1–2 false positive findings since we did not correct for multiple testing and used a significance level of *P* < 0.05. We chose to do so, because this was the first study of its kind and mainly served as exploratory. Fourth, our sample consisted of parents from the Netherlands and Belgium, but only Dutch reference data were available. Due to small sample size, we were unable to correct for country of residence.

## Conclusions

This is the first study that assessed parental distress, including fathers, and everyday problems in parents of children with new-onset SSNS. Our results show that parents with a child with SSNS do not experience more clinically elevated distress than reference parents at 4 weeks after onset. However, more problems were exhibited in parenting problems as well as several everyday problems, the latter being present in both mothers and fathers. How parental distress develops over time in the presence or absence of relapses warrants further investigation. The results of the longitudinal data of the LEARNS study (up to 2 years of follow-up after onset) are awaited. In the meantime, clinicians are encouraged to monitor parental distress using ParROMs to timely identify symptoms and offer support where needed.

### Supplementary Information

Below is the link to the electronic supplementary material.

Graphical Abstract (PPTX 109 KB)

Supplementary file2 (PDF 376 KB)

## Data Availability

The datasets generated during and/or analyzed during the current study are available from the corresponding author on reasonable request.

## References

[1] Rovin BH, Adler SG, Barratt J, Bridoux F, Burdge KA, Chan TM, Cook HT, Fervenza FC, Gibson KL, Glassock RJ, Jayne DRW, Jha V, Liew A, Liu ZH, Mejía-Vilet JM, Nester CM, Radhakrishnan J, Rave EM, Reich HN, Ronco P, Sanders JSF, Sethi S, Suzuki Y, Tang SCW, Tesar V, Vivarelli M, Wetzels JFM, Floege J (2021). KDIGO 2021 clinical practice guideline for the management of glomerular diseases. Kidney Int.

[2] Noone DG, Iijima K, Parekh R (2018). Idiopathic nephrotic syndrome in children. Lancet.

[3] Tarshish P, Tobin JN, Bernstein J, Edelmann CM (1997). Prognostic significance of the early course of minimal change nephrotic syndrome: report of the International Study of Kidney Disease in Children. J Am Soc Nephrol.

[4] Bronner MB, Peek N, Vries M, Bronner AE, Last BF, Grootenhuis MA (2009). A community-based survey of posttraumatic stress disorder in the Netherlands. J Trauma Stress.

[5] Hatzmann J, Heymans HS, Ferrer-i-Carbonell A, van Praag BM, Grootenhuis MA (2008). Hidden consequences of success in pediatrics: parental health-related quality of life–results from the Care Project. Pediatrics.

[6] Streisand R, Braniecki S, Tercyak KP, Kazak AE (2001). Childhood illness-related parenting stress: the pediatric inventory for parents. J Pediatr Psychol.

[7] Barakat LP, Patterson CA, Weinberger BS, Simon K, Gonzalez ER, Dampier C (2007). A prospective study of the role of coping and family functioning in health outcomes for adolescents with sickle cell disease. J Pediatr Hematol Oncol.

[8] Cousino MK, Hazen RA (2013). Parenting stress among caregivers of children with chronic illness: a systematic review. J Pediatr Psychol.

[9] Haverman L, Van Oers HA, Limperg PF, Houtzager BA, Huisman J, Darlington AS, Maurice-Stam H, Grootenhuis MA (2013). Development and validation of the distress thermometer for parents of a chronically ill child. J Pediatr.

[10] Wallander JL, Varni JW (1998). Effects of pediatric chronic physical disorders on child and family adjustment. J Child Psychol Psychiatry.

[11] Mitra S, Banerjee S (2011). The impact of pediatric nephrotic syndrome on families. Pediatr Nephrol.

[12] Mishra K, Ramachandran S, Firdaus S, Rath B (2015). The impact of pediatric nephrotic syndrome on parents’ health-related quality of life and family functioning: an assessment made by the PedsQL 4.0 family impact module. Saudi J Kidney Dis Transplant.

[13] Esezobor CI, Solarin AU, Olagunju AT (2020). Significant burden and psychological distress among caregivers of children with nephrotic syndrome: a cross-sectional study. Can J Kidney Health Dis.

[14] Vance JC, Fazan LE, Satterwhite B, Pless IB (1980). Effects of nephrotic syndrome on the family: a controlled study. Pediatrics.

[15] Veltkamp F, Khan DH, Reefman C, Veissi S, Van Oers HA, Levtchenko E, Mathôt RAA, Florquin S, Van Wijk JAE, Schreuder MF, Haverman L, Bouts AHM (2019). Prevention of relapses with levamisole as adjuvant therapy in children with a first episode of idiopathic nephrotic syndrome: study protocol for a double blind, randomised placebo-controlled trial (the LEARNS study). BMJ Open.

[16] Haverman L, Van Oers HA, Limperg PF, Hijmans CT, Schepers SA, Sint Nicolaas SM, Verhaak CM, Bouts AHM, Fijnvandraat K, Peters M, Van Rossum MA, Van Goudoever JB, Maurice-Stam H, Grootenhuis MA (2014). Implementation of electronic patient reported outcomes in pediatric daily clinical practice: the KLIK experience. Clin Pract Pediatr Psychol.

[17] van Oers HA, Schepers SA, Grootenhuis MA, Haverman L (2017). Dutch normative data and psychometric properties for the Distress Thermometer for Parents. Qual Life Res.

[18] Cohen J (1992). A power primer. Psychol Bull.

[19] IBM Corp (2021). IBM SPSS Statistics for Windows.

[20] R Core Team (2020). R: a language and environment for statistical computing.

[21] Veltkamp F, Rensma LR, Bouts AHM (2021). Incidence and relapse of idiopathic nephrotic syndrome: meta-analysis. Pediatrics.

[22] Hatzmann J, Peek N, Heymans H, Maurice-Stam H, Grootenhuis M (2014). Consequences of caring for a child with a chronic disease: employment and leisure time of parents. J Child Health Care.

[23] Conijn T, Nijmeijer SCM, van Oers HA, Wijburg FA, Haverman L (2019). Psychosocial functioning in parents of MPS III patients. JIMD Reports.

[24] Krom H, van Oers HA, van der Sluijs VL, van Zundert SMC, Otten MAGM, Haverman L, Benninga MA, Kindermann A (2021). Health-related quality of life and distress of parents of children with avoidant restrictive food intake disorder. J Pediatr Gastroenterol Nutr.

[25] Marchal JP, van Oers HA, Maurice-Stam H, Grootenhuis MA, van Trotsenburg ASP, Haverman L (2017). Distress and everyday problems in Dutch mothers and fathers of young adolescents with Down syndrome. Res Dev Disabil.

[26] van Oers HA, Haverman L, Olieman JF, Neelis EG, Jonkers-Schuitema CF, Grootenhuis MA, Tabbers MM (2019). Health-related quality of life, anxiety, depression and distress of mothers and fathers of children on home parenteral nutrition. Clin Nutr.

[27] Diederen K, Haverman L, Grootenhuis MA, Benninga MA, Kindermann A (2018). Parental distress and quality of life in pediatric inflammatory bowel disease: implications for the outpatient clinic. J Pediatr Gastroenterol Nutr.

[28] Warnink-Kavelaars J, van Oers HA, Haverman L, Buizer AI, Alsem MW, Engelbert RHH, Menke LA (2021). Parenting a child with Marfan syndrome: distress and everyday problems. Am J Med Gen A.

