# Supplementary material for: Distress in parents of children with first-onset steroid-sensitive nephrotic syndrome
Source: Pediatr Nephrol. 2023 Jun 28;38(12):4013–22. doi: 10.1007/s00467-023-06038-1 (PMC10584702; doi:10.1007/s00467-023-06038-1)
Supplement: Supplementary file 1 — Graphical Abstract (PPTX 109 KB) [file 467_2023_6038_MOESM1_ESM.pptx]

## Slide 1
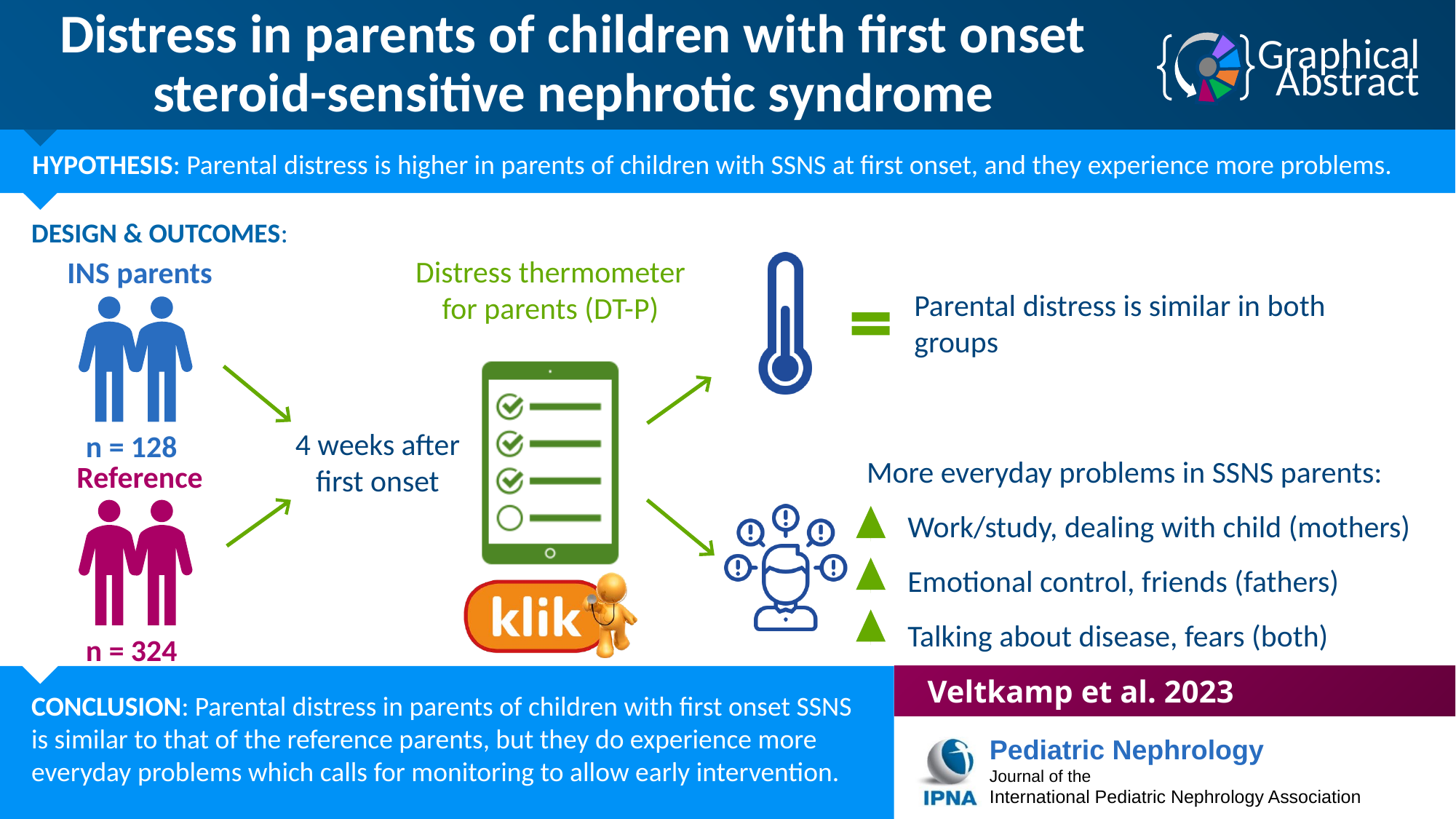

Distress in parents of children with first onset steroid-sensitive nephrotic syndrome
HYPOTHESIS: Parental distress is higher in parents of children with SSNS at first onset, and they experience more problems.
DESIGN & OUTCOMES:
Distress thermometer for parents (DT-P)
INS parents
Parental distress is similar in both groups
4 weeks after first onset
n = 128
More everyday problems in SSNS parents:
Work/study, dealing with child (mothers)
Emotional control, friends (fathers)
Talking about disease, fears (both)
Reference
n = 324
Veltkamp et al. 2023
CONCLUSION: Parental distress in parents of children with first onset SSNS is similar to that of the reference parents, but they do experience more everyday problems which calls for monitoring to allow early intervention.
