# Supplementary material for: Distress in parents of children with first-onset steroid-sensitive nephrotic syndrome
Source: Pediatr Nephrol. 2023 Jun 28;38(12):4013–22. doi: 10.1007/s00467-023-06038-1 (PMC10584702; doi:10.1007/s00467-023-06038-1)
Supplement: Supplementary file 2 — Supplementary file2 (PDF 376 KB) [file 467_2023_6038_MOESM2_ESM.pdf]

Online Resource 1

Supplemental Material

to:

Distress in parents of children with first onset steroid-sensitive nephrotic syndrome.

Floor Veltkamp[1,2], Hedy A van Oers[2,3,4], Lorynn Teela[2,3,4], Elske M Mak-Nienhuis[1], Lotte Haverman[2,3,4]†, Antonia HM Bouts[1,2]†*, on behalf of the LEARNS consortium‡.

† Contributed equally as co-senior authors.

‡ A complete list of study group members appears in the Acknowledgments.

[1] Amsterdam University Medical Centers location University of Amsterdam, Emma Children’s Hospital, Department of Pediatric Nephrology, Meibergdreef 9, Amsterdam, the Netherlands.
[2] Amsterdam Reproduction and Development, Child development, Amsterdam, The Netherlands.
[3] Amsterdam University Medical Centers, location University of Amsterdam, Emma Children’s Hospital, Child and Adolescent Psychiatry & Psychosocial Care, Meibergdreef 9, Amsterdam, The Netherlands.
[4] Amsterdam Public Health, Mental health, Amsterdam, the Netherlands.

Corresponding author: Antonia H.M. Bouts, MD PhD, Amsterdam University Medical Centers, University of Amsterdam, Emma Children’s Hospital, Department of Pediatric Nephrology, Post box 22660, 1100 DD, Amsterdam, the Netherlands.
a.h.bouts@amsterdamumc.nl

Table S1. Cronbach's *α* of the subdomain scores and total scores of the Distress Thermometer for Parents. A subdomain with an *α* < 0.500 is considered to have low internal consistency and scores from thereof should, therefore, not be presented.

	**SSNS**		**Reference**	
	**Mothers**	**Fathers**	**Mothers**	**Fathers**
	α	***α***	***α***	***α***
**Practical problems**	0.713	**0.413**	0.595	0.590
**Social problems**	0.761	**0.181**	0.525	**0.472**
**Emotional problems**	0.746	0.755	0.793	0.766
**Physical problems**	0.600	0.617	0.690	0.640
**Cognitive problems**	0.814	**0.315**	0.732	0.603
**Parenting problems**	0.535[a]	0.600	0.644[a]	0.726[a]
**Total score (5 domains)**	0.883	0.834	0.878	0.862
**Total score (6 domains)**	0.884[a]	0.845	0.885[a]	0.875[a]

[a] Parents of a child ≥2 years of age wrongfully indicated that their child was <2 years of age. As a result, 2, 7, and 10 parents from the SSNS mother, reference mother, and reference fathers, respectively, did not complete the domain Parenting problems for children ≥2 years of age.

Table S2. Baseline characteristics of children with SSNS at four weeks after first onset.

	SSNS children (N=40)
**Country of residence, n (%)**	
Belgium	5 (12.5)
Netherlands	35 (87.5)
**Age (years), median (min, max)**	6.34 (2.6, 15.5)
**Male, n (%)**	25 (62.5)
**Time to remission (days), median (min, max)**	10 (5, 30)
Late remission (>10 days after start treatment), n (%)	20 (50.0)
**Prescribed prednisolone dose (mg/dose), mean (SD)**	51.0 (9.4)
**Cumulative prednisolone dose (mg), mean (SD)**	1590 (354)
**Cumulative prednisolone dose (mg/m2), mean (SD)**	1890 (667)
**Illness in past 4 weeks, n (%)**	11 (27.5)
Days of illness, median (min, max)[b]	2 (1, 10)
Illness for >1 week, n (%)[b]	1 (2.5)
**School absence in past 4 weeks, n (%)**	14 (35.0)
Days of school absence, median (min, max)[b]	10 (1, 28)
School absence for >1 week[b]	8 (20.0)
**Presence of steroid side-effects, n (%)**	34 (85.0)
Binge eating, n (%)	25 (62.5)
Moon face, n (%)	22 (55.0)
Behavioral changes, n (%)	17 (42.5)
Mood changes, n (%)	18 (45.0)
Weight gain, n (%)	12 (30.0)
**Use of concomitant medication, n (%)**	35 (87.5)
Number of used medications, mean (SD)	2.25 (1.1)
Vitamin D, n (%)	34 (85.0)
Proton pump inhibitors, n (%)	11 (27.5)
Antihypertensive medication, n (%)	4 (10.0)
Inhalation medication, n (%)	3 (7.5)
Antibiotics, n (%)	3 (7.5)
Allergy medication, n (%)	2 (5.0)
Other, n (%)	11 (27.5)

[a] Low: primary education, lower vocational education, lower or middle general secondary education; Intermediate: middle vocational education, higher secondary education, pre-university education; High: higher vocational education, university.
[b] Proportion of children who were ill or missed school in the past 4 weeks.

Table S3. Results from the univariate regression analysis assessing the association between sociodemographic and clinical variables and outcome. Variables that were significantly associated with outcome were included in the final multiple regression model.

	Thermometer score		Practical problems		Social problems		Emotional problems		Physical problems		Cognitive problems		Parenting problems		Total (5 domains)		Total (6 domains)	
	**β**	***p***	**β**	***p***	**β**	***p***	**β**	***p***	**β**	***p***	**β**	***p***	**β**	***p***	**β**	***p***	**β**	***p***
**Age parent (years)**	-0.13	0.32	**-0.30**	**0.017**	-0.18	0.16	0.04	0.78	-0.07	0.58	-0.21	0.09	-0.21	0.11	-0.16	0.23	0.06	0.69
**Father**	0.03	0.80	0.15	0.24	-0.01	0.92	-0.02	0.91	0.00	0.98	0.11	0.37	0.19	0.15	0.05	0.69	0.12	0.46
**Born outside NL/BE**	-0.11	0.41	-0.04	0.76	-0.18	0.16	-0.01	0.93	0.09	0.51	0.07	0.60	-0.10	0.46	-0.01	0.93	0.01	0.97
**High educational level**	-0.17	0.22	0.04	0.76	0.03	0.83	-0.12	0.38	-0.01	0.96	0.03	0.82	0.13	0.33	-0.03	0.83	-0.11	0.51
**Paid employment**	-0.11	0.41	0.17	0.19	-0.05	0.71	-0.10	0.46	0.09	0.50	0.07	0.58	0.13	0.33	0.04	0.78	-0.10	0.54
**Number of children (n)**	-0.19	0.14	-0.06	0.65	-0.12	0.34	-0.16	0.22	-0.17	0.20	-0.06	0.64	-0.11	0.40	-0.16	0.22	-0.21	0.18
**Age child (years)**	0.14	0.27	0.11	0.39	-0.21	0.10	0.21	0.10	0.23	0.07	-0.08	0.52	-0.12	0.37	0.14	0.29	0.10	0.54
**Male child**	**-0.26**	**0.039**	-0.09	0.47	-0.05	0.72	-0.15	0.24	0.08	0.55	-0.06	0.65	0.04	0.75	-0.08	0.51	-0.15	0.33
